# Chinese Sausage Simulates High Calorie–Induced Obesity In Vivo, Identifying the Potential Benefits of Weight Loss and Metabolic Syndrome of Resveratrol Butyrate Monomer Derivatives

**DOI:** 10.1155/jnme/8414627

**Published:** 2025-05-16

**Authors:** Ping-Hsiu Huang, Yu-Wei Chen, Cheng-Kai Shie, Shin-Yu Chen, Bao-Hong Lee, Li-Jung Yin, Chih-Yao Hou, Ming-Kuei Shih

**Affiliations:** ^1^School of Food, Jiangsu Food and Pharmaceutical Science College, No. 4, Meicheng Road, Higher Education Park, Huai'an, Jiangsu 223003, China; ^2^Department of Food Science and Biotechnology, National Chung Hsing University, Taichung 40227, Taiwan; ^3^Department of Pediatrics, Kaohsiung Chang Gung Memorial Hospital, Kaohsiung 83301, Taiwan; ^4^Department of Seafood Science, College of Hydrosphere, National Kaohsiung University of Science and Technology, Kaohsiung 81157, Taiwan; ^5^Department of Food Science, National Pingtung University of Science and Technology, Pingtung 91201, Taiwan; ^6^Department of Horticulture, National Chiayi University, Chiayi 600355, Taiwan; ^7^Graduate Institute of Food Culture and Innovation, National Kaohsiung University of Hospitality and Tourism, Kaohsiung 812301, Taiwan

**Keywords:** dysbiosis, metabolic syndrome, obesity, PPAR-γ, resveratrol butyrate esters

## Abstract

This study examined the health benefits of 3-O-butanoylresveratrol (ED4), a monoester derivative of resveratrol butyrate esters. Using a high-calorie diet model simulation with Chinese sausage, ED4 was tested against changes in physiological indices like body weight (BW), body fat, blood pressure, and SCFA levels (stools and serum) in rats. This study found that the obesity-inducing model utilizing sausage as a high-calorie diet worked, and that supplementing rats with ED4 (20 mg/kg BW/day) for 5 weeks inhibited BW increase and body fat buildup. Blood lipid and SCFA dysregulation improved significantly. In addition, ED4 effectively increased PPAR-γ and decreased SREBP-1C mRNA expression, preventing fat accumulation and overproduction. A novel food-driven relationship between gut microbiota and adipose was found, promoting health. Our findings showed that ED4 supplementation exacerbated metabolic abnormalities caused by high-calorie diets and reduced body fat. Notably, these metabolic benefits were enhanced through the involvement of intestinal microbiota.

## 1. Introduction

Considering the global prevalence of obesity and its associated metabolic disorders, which pose significant challenges to both developing and developed countries, the inability to entirely consume delicious meals has long been a persistent issue for humanity [1, 2]. Accumulated data from earlier studies have demonstrated the correlation between gut microbiota composition and metabolic diseases, which can be a practical approach for preventing and treating obesity and related metabolic disorders [2–5]. Relevant research and investment in intestinal microbiota have garnered significant attention recently. The interactions between gut microbiota and the host are complex, wherein introduced strains colonize the gut and modify the metabolic environment by reshaping intestinal bacterial communities and producing metabolites that regulate host physiology and maintain metabolic homeostasis [2]. Indeed, the implication for various lifestyles could be that the relatively stable distribution of the gut microbiota can generate fluctuations [5–7]. Despite extensive research into probiotics and prebiotics, while necessary, the potential for long-term supplementation has a positive impact on the restructuring of gut microflora, thereby contributing to BW management and overall health [6, 8–10]. However, it is imperative to acknowledge that weight loss poses a significant challenge, mainly because of the multifaceted and intricate underlying causes. Integrating BW loss solution features into palatable food products, without relying on extra approaches, may enhance consumer acceptance while serving as a strategic measure contributing to successful BW loss.

The novel derivatives of resveratrol (RSV), known as RSV-butyrate esters (RBEs), have been previously synthesized by this team using the butyrate acid esterification method [11–13]. Among the various structural monomers, two distinct compounds are ED2 (3,4′-di-O-butanoylresveratrol) and ED4 [12, 13]. These substances have been widely reported to inhibit lipid accumulation in the liver [4, 12], interrupt the conversion of trimethylamine (TMA) to trimethylamine N-oxide (TMAO) [14], prevent cardiovascular disease [5, 13, 15], avoid hypertension because of chronic kidney disease [16–19], and inhibit the development of obesity caused by bisphenol A in female rats offspring [20], apart from acting as cellular antioxidants [10–13] (in vivo and in vitro studies). In addition, in several of the above studies, our team also observed that these RSV derivatives (RBE, ED2, and ED4) can regulate the dysbiosis of the intestinal microbiota towards regaining homeostasis and mitigate these adverse health risks such as metabolic diseases such as obesity, disrupted lipid profiles, or hypertension, via SCFA formations [4, 8, 16–20]. Remarkably, combined with the team's food processing expertise, RSV, ED2, and ED4 (500 ppm) have been successfully applied in Chinese sausage manufacturing as dietary supplements recently, preventing lipid oxidation apart from the reduction of sodium nitrite (only 25 mg/kg sodium nitrite is requested), while the antimicrobial effect is prolonged in storage for at least 30 days, namely, providing an beneficial antioxidant product for people of all ages [10].

Moreover, in the previous procedure of inducing obesity in rats, we observed that despite the successful induction of obesity through a high-fat diet (Research Diet D12451) [4], it is worth acknowledging that the food intake in the induced group of rats was approximately 22.50% lower compared to that in the control group. Consequently, building on the model proposed by Crowe et al. [21], which aims to approximate reality while better reflecting real-world human food choices, this study integrates existing research findings to establish and validate a high-calorie diet model simulation using Chinese sausage. Furthermore, by leveraging our team's expertise in food processing, we have addressed the reported limitations of this diet, specifically the challenge of precisely matching nutritional properties with isocaloric equivalents.

Therefore, this study used the high-fat profile of Chinese sausage to develop and validate a high-calorie diet model. It examines physiological parameters such as growth performance, SCFA, serum lipid profiles, and fat accumulation related to obesity in rats exposed to a high-calorie diet. In addition, this study also considered the composition and regulation of the intestine microflora in looking for solutions that effectively combat obesity, manage BW, and advocate for avoiding adipose accumulation. In light of the accumulation of previous results with sufficient evidence, we hypothesize that continuous supplementation of ED4 facilitates BW loss, regulation of serum components, and recovery of intestinal microbiota steady-state in rats.

## 2. Materials and Methods

### 2.1. Materials

Lean meat and back fat of pork were purchased from Master Channels Co. (Tainan City, Taiwan). Food-grade ingredients, including nitrite, sugar, and salt, were procured from a local supermarket (Kaohsiung, Taiwan). RSV (99%) was purchased from Tokyo Chemical Industry Co., Ltd. (Chennai, Tamil Nadu, India). All the chemicals were purchased from Chem Service Inc. (West Chester, PA, USA) and used without any extra steps unless stated otherwise. The samples of RSV and ED4 were prepared following the previously published protocols established by this team [10, 12, 20].

### 2.2. Chinese Sausage Simulates a High-Calorie Diet

The recipe and process of Chinese sausages were modified based on our team's previously published research [10]. All ingredients (Supporting Table A1) were mixed evenly, freeze-dried, milled into powder, and vacuum-packed. Afterward, a high-calorie diet feed was prepared by mixing purified rodent diet (AIN-76A) with the above-mentioned Chinese sausage powder at a ratio of 85:15 (w/w), as described in Crowe et al. [21]. The nutritional composition of the feed is shown in Supporting Table A2) the above-mentioned Chinese sausages powder. Subsequently, the feeds were freeze-dried for setting. Considering the risk of mitigating lipid oxidation in the sausages, all feeds were vacuum-packed, then stored at −80°C as in the case of AIN-76A, and prepared weekly during the experimental period.

### 2.3. Experimental Animal and Design

The animal experiments in this study was performed as described by Chen et al. [4], with minor modifications. A total of 24 male, 4-week-old Sprague-Dawley (SD) rats were purchased from BioLASCO Taiwan Co., Ltd. (Taipei, Taiwan), while the average initial BW ranges from 89.9 to 100 g. The rats were housed in cages containing stainless steel suspended wire mesh crates (two rats per cage) within an environment free from specific pathogens. The temperature was maintained at 22 ± 2°C, and humidity levels were 55 ± 5%, followed by a 12 h light/12 h dark cycle, with lights being turned on at 7 p.m. They were provided *ad libitum* access to diet (AIN-76A) and water throughout the experiment. The feed was replenished daily, while the ambient drinking water (sterilized at 121°C for 15 min) and bedding were refreshed every 3 days, all in the morning. After a 1-week adaptation period (BW of all rats reached 150 g), the four groups of six rats each (*n* = 6) were randomly assigned as follows: control diet (CN) group and three groups of rats on Chinese sausage in high-calorie diet (SHD). Among them, two groups were treated with RSV (20 mg/kg BW/day; SHDR) and ED4 (20 mg/kg BW/day; SHDM), respectively.

**Figure 1:**
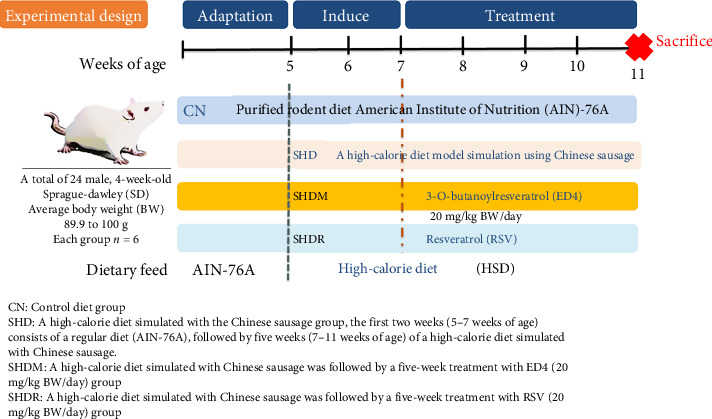
Validation of a high-calorie diet model simulation using Chinese sausage experimental design.

This study was performed for 7 weeks (Figure 1); the first 2 weeks (5–7 weeks of age) were obesity-inducing, and the subsequent 5 weeks (7–11 weeks of age) evaluated the effects of RSV and ED4 on BW management and physiological parameters. Specifically, the SHD group was given a regular diet (AIN-76A) for the first 2 weeks, followed by a Chinese sausage-simulated high-calorie diet for the remaining 5 weeks. For the SHDR and SHDM groups, the Chinese sausage was utilized to simulate a high-calorie diet provided throughout the entire duration, except that tube-feeding of 20 mg/kg BW/day of RSV or ED4 was administered daily during the last five weeks. All operations and analyses during the experimental period are described in the subsequent sections, while at the endpoint of the experiment (11 weeks of age), all rats were fasted for 24 h before sacrifice. The SD rats were anesthetized by intraperitoneal injection with a mixture of 25 mg/kg Zoletil (50, Virbac Laboratories, Carros, France) and 23.32 mg xylazine hydrochloride/kg Rompun (Dechra Pharmaceuticals PLC., Lostock Gralam, UK) at a 1:1 ratio (v/v). Immediately, cardiac blood sampling was performed, and blood was collected in disposable vacuum blood collection containers for subsequent analysis. All organs were weighed, and a fraction of the liver, subcutaneous tissue, peritoneal fats, and ileum was immersed in 10% formalin for subsequent histological analysis. The remaining portion and other tissues were frozen with liquid nitrogen and preserved at −80°C for subsequent analysis.

The animal procedures followed the guidelines for the Care and Use of Experimental Animals established by the Committee for the Purpose of Control and Supervision of Experiments on Animals and the National Institutes of Health. The protocol under code 0110-AAAP-012 was approved by the Committee on Animal Research at the National Kaohsiung University of Science and Technology.

### 2.4. Evaluation of the Performance of Rats in Terms of Growth

During the experiment, rats' diet, water intake, and BW were recorded daily or weekly for observation and evaluation of the developmental status of the rats. The evaluated parameters encompassed the BW change curve, mortality, feed conversion rate (FCR), specific growth rate (SGR), and relative organ relative weights. Moreover, the blood pressure (BP) of rats in each group was measured using a noninvasive BP analyzer (Softron BP-2010A, Softron Biotechnology Co. Beijing, China) performed according to standard operating procedures (SOPs) provided by the manufacturer. The systolic BP (SBP) and diastolic BP (DBP) were determined by analyzing the changes in the amount of light transmitted by the tail vasodilatation (equivalent to contraction). Briefly, BP was measured in the rat's tail using a tail-sleeve sensor, with each measurement lasting for 10–15 s. The baseline BP measurement was essential during the adaptation phase to establish a reference point for guaranteeing the precision of subsequent experimental outcomes.

### 2.5. Measurement of Serum Lipoprotein Composition

There were three blood sampling programs, whereby blood samples were collected from the tails of all animals during the two previous blood sampling programs (including the 5 to 7 weeks of age during the obesity induction period). The last blood collection was conducted following the sacrifice of an animal, and the blood was immediately centrifuged at 4°C and 1500 × *g* for 10 min using a microfuge (CF 16RN, Hitachi, Ltd., Tokyo, Japan) while the serum was stored at −80°C for subsequent analysis. All measurements were conducted under the manufacturer's SOPs. The total cholesterol (TC), triglyceride (TG), and high-density lipoprotein (HDL) contents were analyzed using an automated clinical chemistry analyzer (NX-500i, Fuji, Masaaki Takei, Japan). Furthermore, the low-density lipoprotein (LDL) and very low-density lipoprotein (VLDL) contents were analyzed using the ELISA kits (CSB-E11704r and E17088r, CUSABIO Technology LLC., Houston, TX, USA). Briefly, 5 μL of serum followed by 50 μL of diluent and 50 μL of HRP-conjugate were added to a 96-well dish. The reaction was then carried out at 37°C for 30 min to avoid the light. Subsequently, all solutions were removed, and the wells were washed five times with 200 μL wash buffer. Afterward, 90 μL of TMB-substrate was added and incubated for 20 min at 37°C under light-protected conditions, followed by 50 μL of stop solution. The samples' absorbance values were measured at a wavelength of 450 nm using an ELISA reader (BioTek, Agilent Technologies, Inc., Santa Clara, CA, USA), with a reference wavelength set at 540 nm for accurate correction.

### 2.6. Histological Analysis

The histological analysis of the liver and subcutaneous and peritoneal fat tissues was conducted using the protocol outlined by Huang et al. [22], with minor modifications. The tissues were fixed in 10% formalin for 24 h. Subsequently, the tissues were dehydrated using a series of level alcohols (70%, 80%, 90%, and 95%) for 2 min each, followed by clearing in xylene. The fixed tissues were embedded in paraffin, sectioned into 5 μm thick slices, and stained with hematoxylin and eosin (H&E). Images of the stained slices were captured using a MoticEasyScan system and analyzed using the Motic DS Assistant (4K).

### 2.7. Measurement of Short-Chain Fatty Acids in Serum and Stool

The short-chain fatty acids measurements in serum and stool were performed according to the method described by Chen et al. [4]. The rats' stool samples weighing 0.1 g were homogenized in 1 mL of double-distilled water (DDW), followed by centrifugation (9000 × *g* at 4°C) for 10 min and subsequent collection of the supernatant. Then, 150 μL of the sample (serum or stool supernatant) was acidified with 50 μL sulfuric acid. After adding 10 μL of 2-ethylbutyric acid as the internal standard, After adding 10 μL of 2-ethylbutyric acid as the internal standard, the mixture was vortexed for 15 minutes with 400  μL of diethyl ether. Afterward, the mixture underwent centrifugation of 9000 × *g* at 4°C for 10 min, while the resulting liquid above the sediment was collected for analysis using gas chromatography-mass spectrometry (GC–MS). The detailed conditions were as follows: The chromatographic analysis employed a DB-FFAP capillary column (30 m × 0.25 mm × 0.25 μm), with the mobile phase gas (helium) delivered at a controlled flow rate of 1 mL/min. The heating program was conducted as follows: the temperature was initially set at 80°C and maintained for 1 min, then gradually increased to 240°C at 10°C/min and sustained for 12 min. The temperature settings for the injector, transfer line, ion source (set at 70 eV), and the quadrupole were 240, 230, 150, and 150°C, respectively. The scanning range was optimized to encompass a mass-to-charge ratio (m/z) ranging from 35 to 550. The identification of the compound type was substantiated through a comparative analysis of the compound's mass spectrum with the GC–MS database.

### 2.8. Determination of Lipid-Regulated Genes and Protein Expression in the Liver

The liver tissue 100 mg was added to 250 μL of Trizol and broken using a ball mill (Fisher Scientific Bead Mill 4, Thermo Fisher Scientific) (Mode 3, total of 3 times for 15 s each). Next, 750 μL of Trizol and 250 μL of chloroform were added, and vortex-induced oscillation for 15 s was performed on ice for 5 min, followed by centrifugation (4°C, 6000 × *g*) for 10 min. The supernatant was collected and transferred to a new Eppendorf, and then an equal volume of isopropanol was added for mixing uniformly and allowed to stand on ice for 5 min for the RNA to precipitate before centrifugation (4°C, 6000 × *g*) for 15 min. Next, the supernatant was separated, and 1 mL of 70% ethanol was added, followed by centrifugation (4°C, 6000 × *g*) for 10 min. This process was repeated twice to remove the organic solvents. Finally, the residue (RNA) was air-dried at 25°C, and DEPC-H2O was added. Then, the ratio of absorbance at 260/280 nm (in the range of 1.6–1.8) was measured using a microplate reader (Bio Tek-EPOCH 2 Agilent Technologies Inc., Santa Clara, CA, USA), and the sample concentration was calculated to confirm the total RNA quality. Subsequently, using the cDNA reagent kit (GeneDireX, Inc., Taoyuan, Taiwan), add 1 μL of Oligo(dT) 20, 1 μL of dNTP mix, 10 μL of RNA, followed by sterile water to make a volume of 13 μL in a 0.2 mL PCR tube. Next, transfer to a dry bath at 65°C for 5 min, perform vortex-induced oscillation, and place on ice for 5 min, then add 4 μL of 5X first strand buffer, 1 μL of DTT, 0.25 μL of RiboINTM RNase inhibitor, and 1 μL of GScript RTase, before adding sterile water to make a volume of 20 μL. Afterward, the solution was dry bathed at 50°C for 1 h, followed by 70°C for 15 min, while the resulting cDNA solution was stored at −20°C until it was used.

In the 96-well plate for PCR, 7 μL of 2 × SYBR Fast MM, 2 μL of 20× Target primer set (Supporting Table A3), 6 μL of DDW, and 2 μL of cDNA (4000 ng/20 mL) were added to make a total volume of 17 μL. This study selected r18s with constant specific gene expression in mice as the control group for relative quantification. Afterward, it was placed into a 96 Real-Time PCR system (LightCycler, F. Hoffmann-La Roche Ltd, Basel, Switzerland) for reaction and fluorescence quantification (conditions as shown in Supporting Table A4).

### 2.9. Next-Generation Sequencing (NGS) Analysis and Bioinformatics of Intestinal Flora

Rat stool samples were collected, preprocessed, analyzed, and subjected to bioinformatic analysis following the description of Chen et al. [4]. Rat stool samples were collected (the day prior to sacrifice), immersed in a preservation solution, stabilized, and subsequently frozen at −80°C for subsequent analysis of intestinal microbiota. In this study, the Quick-DNA Fecal/Soil Microbe Miniprep kit (Zymo BIOMICS, Zymo Research Co., Irvine, CA, USA) was employed for bacterial deoxyribonucleic acid (DNA) extraction. The concentration (absorbance wavelength ratio of 260/280 should fall within the range of 1.7–2.2, while the concentration should be higher than 50 ng/μL) was determined using a spectrophotometer (NanoDrop 2000, Thermo Fisher Scientific, Waltham, MA, USA), and the DNA was subsequently diluted by a factor of 10 to achieve a final concentration of 4–6 ng/μL using an elution buffer. Subsequently, Biotools Co., Ltd. (Taipei, Taiwan) was commissioned to prepare DNA libraries through polymerase chain reaction (PCR) amplification of the highly variable V3–4 regions of the 16S ribosomal RNA (rRNA) gene. The sequencing was conducted using a Miseq paired-end reader platform (Illumina Inc., San Diego, CA, USA), in which each sample generated paired-end reads (2 × 300 nt) with a minimum of 100,000 reads. The initial paired-end reads underwent trimming, and those that met the quality criteria were categorized into operational taxonomic units (OTUs) with a similarity of ≥ 97% to entries in the training dataset for error rates and sample inference. This categorization was used to identify amplicon sequence variants (ASVs), which serve as proxies for species, by comparing them to the National Center for Biotechnology Information (NCBI) 16S rRNA database. The OTU taxonomy analysis, including visualization of relative abundance and heatmap, was performed using Basespace (Illumina Inc.), CLC genomics workbench (Qiagen, Hilden, Germany), and GraphPad Prism 8 software (GraphPad Software, Boston, MA, USA). α diversity was assessed by calculating the Menhinick, Pielou's evenness, and Simpson. β diversity analysis involved Bray-Curtis, principal coordinates analysis (PCA) with Ellipse, and partial least squares discriminant analysis (PLS-DA). The core bacteria analysis was conducted using the linear discriminant analysis effect size (LEfSe), functional analysis was performed using Phylogenetic Investigation of Communities by Reconstruction of Unobserved States (PICRUSt) via the Galaxy/Hutlab [23] website, and the statistical significance was determined at *p* < 0.05.

### 2.10. Statistical Analysis

The data presented in the figures and tables of this study were expressed as the mean ± standard error of the mean (SEM). The data obtained in this study were subjected to statistical analysis using the Statistical Package for the Social Sciences (SPSS) software (V26.0, International Business Machines (IBM) Co., Armonk, NY, USA). The differences were analyzed using a one-way analysis of variance (ANOVA), Tukey's method was used to compare the differences in groups, and *p* < 0.05 was considered a statistically significant difference.

## 3. Results and Discussion

### 3.1. Validation of a High-Calorie Diet Model Simulation Using Chinese Sausage

**Figure 2:**
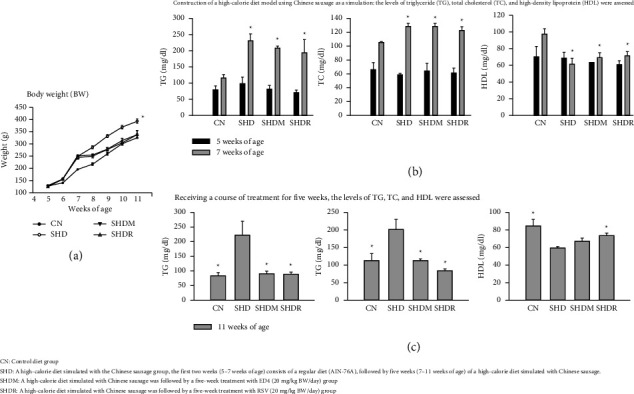
Effects on various serum physiological parameters. (a) Body weight (BW), (b) construction of a high-calorie diet model using Chinese sausage as a simulation: the levels of triglyceride (TG), total cholesterol (TC), and high-density lipoprotein (HDL) were assessed and (c) receiving a course of treatment for 5 weeks, the levels of TG, TC, and HDL were assessed in Sprague–Dawley (SD) rats with obesity induced by a high-calorie diet simulated using Chinese sausage, then treated with resveratrol (RSV) and the purified monomer product 3-O-butanoylresveratrol (ED4).

This study used sausage to simulate a high-calorie diet to model induced obesity in SD rats, which revealed that the BW of rats in SHD, SHDM, and SHDR groups were higher than the control group by 2 weeks of induction (5–7 weeks of age) (Figure 2(a)). Subsequently, the same dose (20 mg/kg BW/day) of RSV and ED4 were administered to SHDR and SHDM, respectively, for 5 weeks (7–11 weeks of age). It was found that the BW of the rats in each group was altered and maintained toward the end of the trial, with the maximum BW in the SHD group (*p* < 0.05). In contrast, the BW of the treatment groups (SHDR and SHDM) tended to be similar to that of the CN group, with no statistical difference, while these trends were substantially similar to the findings of the published report [24]. In particular, there was a clear watershed from 8 weeks of age until the end of the trial at 11 weeks. However, these phenomena imply that treating RSV and ED4 effectively controls the weight gain of SD rats on a high-calorie diet. In addition, the BW change in the SHD group of this study was consistent with the results reported by Crowe et al. [21], despite the slight difference in the calorie content of the modified chow diets (same ratios of 85% AIN-76/15% sausage) used. The minor variation was hypothesized to arise from the disparity in the lean meat-to-fat ratios of the ingredients.

Regarding lipid profiles, TG and TC were higher (*p* < 0.05) in the SHD, SHDM, and SHDR groups than in the CN group after two simulated high-calorie diet inductions in sausage (Figure 2(b)). However, the opposite trend was observed in HDL levels, which were maximized in the CN group (*p* < 0.05). The findings of this study on TG and TC levels were similar to previously published results [21], albeit the study reported that the three modified recipes (pork, sausage, and frankfurter sausage) were substantially higher in TG levels and lower in certain cholesterol esters and phosphatidylcholine. Nevertheless, following five weeks of treatment with RSV and ED4, TG and TC in both SHDM and SHDR groups were controlled and close to those of the CN group (Figure 2(c)) and were significantly different (*p* < 0.05) compared to the SHD group. Notably, the TC level in the SHDR group was the lowest of all groups, while both TG and TC levels of SHD were considerably higher than all groups by at least 2-folds. In a study where obesity was induced in C57BL/6 mice using a high-fat diet (5.21 kcal/g, with 60% kcal from fat and 20% kcal from carbohydrates) compared to a Western diet (4.67 kcal/g, with 40% kcal from fat and 43% kcal from carbohydrates), additional supplementation with kefir was provided for ten weeks [25]. The results showed a decrease in TG in the treatment groups, yet they were not significantly different from the high-fat diet versus Western diet-fed groups. These phenomena above exhibited a concordance with the trend uncovered within the parameters of this study. However, it has been reported that the equilibrium of cholesterol metabolism (including production, intake, and elimination) within the body impacts the cholesterol level in the blood [22].

**Figure 3:**
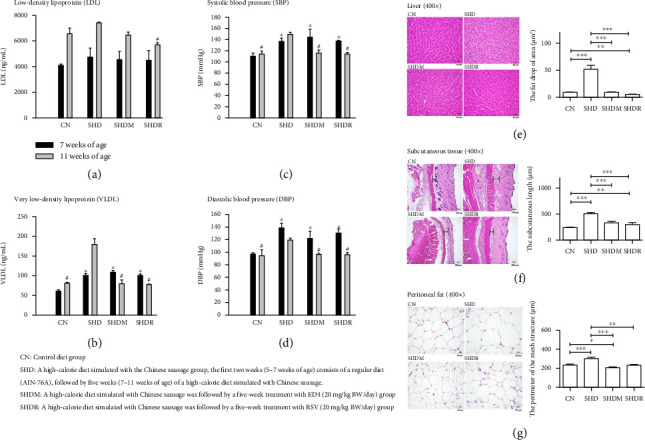
Effects on various serum biochemistry parameters. (a) Low-density lipoprotein (LDL), (b) very low-density lipoprotein (VLDL), blood pressure, (c) systolic blood pressure (SBP), (d) diastolic blood pressure (DBP) and tissue sections, (e) liver, (f) subcutaneous tissue, and (g) peritoneal fat in Sprague–Dawley (SD) rats with obesity induced by a high-calorie diet simulated using Chinese sausage then treated with resveratrol (RSV) and the purified monomer product 3-O-butanoylresveratrol (ED4).

Regarding HDL levels, the SHDR was more similar to the CN group compared to the SHD and SHDM groups (*p* < 0.05), yet HDL levels in the SHDM group were substantially higher than the SHD group. Notably, regular levels of HDL have been widely reported to provide TC transport to the liver for absorption, storage, and metabolism, which supports reduced TC levels in the blood while contributing to a diminished risk of cardiovascular disease [22, 26]. In addition, the levels of other blood lipid biomarkers (including LDL and VLDL) in all rat groups exhibited no significant difference compared to the CN group despite being elevated 2 weeks after obesity induction (at 7 weeks of age) (Figure 3(a)). However, following five consecutive weeks of RSV and ED4 administration, the LDL level was lowest in the SHDR group with a significant difference compared to all groups (*p* < 0.05), and the LDL level was also lower in the SHDM than in the CN group, albeit without significant differences. Similar trends were observed for VLDL levels as for LDL (Figure 3(b)). Specifically, within two weeks of obesity induction, VLDL levels in all SD rat groups were significantly higher than in the CN group (*p* < 0.05). It was predictable that SHDR and SHDM were lower (*p* < 0.05) than the CN group following treatment with RSV and ED4. Interestingly, the SHD group exhibited significantly higher levels of TG, TC, LDL, and VLDL compared to all other groups after 7 weeks of consuming a high-calorie diet, except for a significant decrease in HDL, as mentioned earlier, which was associated with metabolic disorders. Hence, the present study's findings exhibit a consistent trend compared with the previously reported 12 consecutive weeks of supplementation with Naringin (flavanone-7-O-glycoside 100 mg/kg/day, gavage) [27], while this supplementation proved productive in treating obesity induced by a high-fat diet in vivo. Its efficacy reduces the persistent increase in BW under control, reduces body fat accumulation, and contributes to regulating blood lipid biomarkers (including TG, TC, and LDL) levels [27]. Furthermore, it has been reported that high-fat diet-induced obese C57BL/6J male mice supplemented with purified citrus polymethoxyflavone extracts (30, 60, and 120 mg/kg) for eight consecutive weeks showed a pronounced decrease in TC, TG, and LDL, while HDL did not significantly increase [28]. The extracts also had a protective effect against hepatic impairment, while their improvement of metabolic syndrome was dose-dependent [28]. Commonly known as an indicator of atherosclerosis is the HDL/LDL ratio (a high level indicates a high level of HDL and a low level of LDL), suggesting that TC in the blood can be transferred more readily to the liver, thereby assisting in minimizing the risk of atherosclerosis [22]. In addition, the liver LDL receptors play a role in modulating the rate of LDL production and the metabolism of VLDL [22]. It has been reported that hypertension arises from the vascular circuit constriction caused by excessive and prolonged lipid accumulation in the blood, ultimately leading to obstruction, namely, vascular dysfunction [29]. BP refers to the lateral pressure exerted on the vessel wall per unit area during blood flow, whereas hypertension is characterized by an elevation in both systolic and diastolic BP; it has been reported that several of the causes above have a direct impact on BP [30]. It is essential to synchronize the changes in BP (including systolic and diastolic) between different groups of SD rats during the treatment period. Unsurprisingly, this study revealed that BP in the SHD, SHDM, and SHDR groups was higher than in the CN group post-induction, reflecting the earlier observed changes in lipid biomarkers. Specifically, BP parameters were the highest in the SHD group. In contrast, the BP of SHDM and SHDR tended to be closer to that of the CN group following the administration of ED4 and RSV (Figures 3(c) and 3(d)) and were significantly different compared to the SHD group (*p* < 0.05). The high-calorie diet recipe established in this study, including a 15% sausage ratio (with a measurable effect), has been reported as a relatively high processed meat intake for a human diet [21]. Therefore, this study finding suggests that high-calorie diets are linked to an elevated risk of metabolic disorders [31, 32]. Nevertheless, all animals survived the 7-week experimental period without mortality (Supporting Table A5). In addition, FCR for all groups slightly decreased compared to the CN group, with a statistical difference in the SHDM group. It has been reported that modified diets promote increased BW by adding extra ingredients (such as protein, fat, and fiber), which make the feed more appetizing to experimental animals, thereby encouraging them to consume more [21]. In this study, we did not observe any excessive feed consumption in SD rats. Nevertheless, the highest SGR was observed in the SHD group, while no statistical differences were observed between groups. Therefore, in this study, compared to the previously published work by our team (utilizing a high-fat diet Research Diet D12451) [4], employing this modified high-calorie diet model simulation using Chinese sausage proved to be more appropriate for SD rats. This conclusion is supported by significant evidence from observations of BW fluctuations, FCR, and SGR. These phenomena indicated that RSV and ED4 (20 mg/kg BW/day) used in this study did not pose a life-threatening risk to SD rats; thus, immediate toxicity was not significantly evident. However, the safety of consumption (such as the Ames, toxicity, and mutagenicity tests) [33] and genotoxicity can be evaluated via more experimental evidence.

Moreover, the relative organ weights of the left kidney, right kidney, and liver were not substantially different (Supporting Table A5) between the groups, and they were not different visually either. This phenomenon suggested that the high-calorie diet, RSV, and ED4 treatments used in this study could not affect the rats' metabolic mechanisms to cause inflammation in these organs. Remarkably, the SHD group exhibited a significantly higher visceral fat weight (including epididymis and retroperitoneal fats) than the CN group. Conversely, treatment with RSV and ED4 (20 mg/kg BW/day) reduced visceral fat weight, similar to that observed in the CN group. It was hypothesized that these phenomena were attributable to visceral fat accumulation and weight gain in SD rats on a high-calorie diet.

In contrast, five consecutive weeks of treatment with RSV and ED4 successfully reduced the accumulation of epididymis and retroperitoneal fats. Notably, a study has confirmed that early life exposure to a high-fat diet and low-dose penicillin induces elevated fasting blood glucose levels and greater fat accumulation in hepatocytes [32]. The same authors also observed persistent hyperglycemia and a significant increase in BW and abdominal adiposity after discontinuing antibiotics for several weeks. Notably, compared to the published treatment trend of our team using a high-fat diet (Research Diet D12451) [4], the trend for ED4 was consistent and as expected, with satisfactory performance in terms of therapeutic effects, including control of BW, restoration of blood lipid composition and adipogenesis metabolism disorders in obese SD rats. Alternatively, a continuous administration of RSV and ED4 for five consecutive weeks effectively mitigated these adverse effects. These effects may also contribute to BW management and minimize the risk of sustained obesity leading to related diseases such as metabolic syndromes. It was hypothesized that ED4 and RSV possibly provide an effective preventative measure for the development of metabolic syndromes associated with obesity.

### 3.2. Effects on the Morphology of Liver, Subcutaneous Tissue, and Peritoneal Fat

Extensive documentation supports the notion of hepatic stenosis that intracellular fat accumulation progressively leads to fatty liver and chronic inflammation, while the chronic inflammatory state subsequently triggers liver fibrosis and cirrhosis, all exhibiting a strong correlation with obesity and metabolic disorders [22, 34]. This study showed that the liver tissue sections of the SHDM and SHDR groups administered with ED4 and RSV were similar to the CN group (Figure 3(e)), which showed marked improvement compared to the SHD group despite the accumulation of some fatty particles. Mainly, the fat drop in area was highest in the SHD group, whereas it was lowest in the SHDR group, and there were significant differences in each group (*p* < 0.05). This phenomenon indicates that the treatment of ED4 and RSV in this study decreased the size and accumulation of fat drop in the liver. However, the liver serves as the primary storehouse of fat; fat accumulation in the liver occurs because of TG accumulation, while the decrease in the size of lipid droplets in the hepatocytes post-therapy provides evidence of hepatic cellular recuperation [4, 35]. Revealingly, the findings of this study also revealed consistent trends in both subcutaneous tissue and peritoneal fat (Figures 3(f) and 3(g)), mirroring that observed in liver sections, namely, the SHDM and SHDR groups obtained an improvement by ED4 and RSV treatments. Specifically, subcutaneous tissue length was the greatest in the SHD group and the lowest in the CN group, significantly different from each group (*p* < 0.05). However, the SHDM and SHDR groups approached values close to that of the CN group. Nevertheless, the CN group showed a denser mesh structure for peritoneal fat, while the SHDM and SHDR groups improved tissue appearance with treatment, converging with the CN group. As expected, the SHD group exhibited the largest perimeter of the mesh structure, with significant differences among the groups (*p* < 0.05). It has been reported that treatment of high-fat-diet-induced obese mice with *Garcinia indica* extract effectively promotes fatty acid β-oxidation by modulating gut microbiota abundance and contributes to managing BW and restoring regular adipocyte size [36]. Moreover, substantial improvements have been demonstrated in body fat accumulation, lipid deposition in the liver, and cell size of adipocyte (white and brown) tissue in mice induced by a high-fat diet after 8 weeks of continuous administration of a natural substance (polymethoxyflavone) obtained by the purification of citrus [28]. Therefore, all these phenomena in the present study agreed with the results of the published studies mentioned above.

### 3.3. Effects on the Short-Chain Fatty Acid Contents of Serum and Stool

It is well-known that the host can obtain energy from incompletely digested food through the absorption and metabolism of SCFA while also influencing transport and metabolism, growth and differentiation of small intestinal epithelial cells, and controlling lipid and carbohydrate metabolites in liver cells [37]. Specifically, this involves lowering body glucose and TC, altering lipid metabolism, and inhibiting TC synthesis in liver cells, reducing the risk of developing atherosclerotic cardiovascular disease [38].

This study showed a similar trend between SHDM and SHDR groups regarding changes in SCFA levels in the blood (Table 1). In particular, acetic acid content was the lowest in the CN group, followed by the HSDM group, which were significantly different (*p* < 0.05) compared to the others. In addition, the SHDM and SHDR groups exhibited higher levels of propionic and butyric acids than the CN group (*p* < 0.05). In contrast, compared to the HSD group, there were differences in isobutyric and butyric acids (*p* < 0.05). In contrast, the SCFA contents of stool showed significant differences (*p* < 0.05) in all groups compared to the CN group, apart from the propionic acid content (Table 1). Three groups receiving high-fat diets specifically contained 4.0- to 5.5-folds of acetic acids compared to the CN group. It was hypothesized that the predominant SCFAs in the intestines—acetic, propionic, and butyric acids—account for more than 95% of all SCFAs, typically in a 3:1:1 ratio [39, 40]. It has also been demonstrated in human clinical trials that there is a negative correlation between circulating TG levels and the intestinal butyryl-CoA-acetate CoA-transferase pathway, which appears to be the most common pathway for butyrate production in the gut microbiome [31, 41]. The plasma SCFA levels were also more effective, particularly at the acetic, propionic, and butyric acids levels, using purified ED4 in this study compared to previously published works by our team [16]. The association among SCFA levels, gut microbiota, and lipid concentrations in vivo has been validated [31]. However, it has been reported that the intestinal flora responsible for butyrate production include *Ruminococcaceae* spp., *Romboutsia* spp., *Clostridium* spp., *Intestinimonas* spp., *Akkermansia* muciniphila, and other species [42–44], while this study also partially identified these bacteria, as described and discussed in Section 3.5. More evidence will likely continue to be presented as NGS databases are updated and new technologies are developed.

Furthermore, *A. muciniphila* has been explored as a potential next-generation probiotic with a direct correlation between its abundance and metabolic abnormalities (obesity, inflammation, or diabetes), implying that elevating its abundance can benefit health promotion [45–47]. In parallel, it has also been reported that combining *Akkermansia* and probiotics such as *Bifidobacterium* and *Lactobacillus* maintains a healthy intestinal microecology, thereby reducing oxidative stress production in hepatocytes [48]. In addition, the SHDM and SHDR groups were treated with ED4 and RSV administrations, but only the propionic acid levels recovered and converged with those of the CN group. In general, administration of ED4 and RSV effectively restored SCFAs in the blood, while only slight improvement was observed in SCFAs detected in the stool. Furthermore, the SCFAs originating from the gut have been ascertained to influence immune responses in diverse extraintestinal organs, encompassing the liver, lungs, reproductive tract, and brain [49]. These SCFAs have been correlated with therapeutic efficacy in conditions including antibacterial effects, self-immunity, food allergies, autoimmunity, intestinal inflammation, asthma, and cancer [49, 50]. It is well known that apart from the influence of the intestinal microbiota composition, the SCFA composition and content are also affected by dietary intake [50]. It was confirmed by comparison with the published study of this team that despite the similarity of the trend of the composition and contents of SCFAs in the blood and stool, satisfactory performance was demonstrated in the administered ED4 [4].

### 3.4. Effects on the Lipid-Regulated Genes and Protein Expression in the Liver

**Figure 4:**
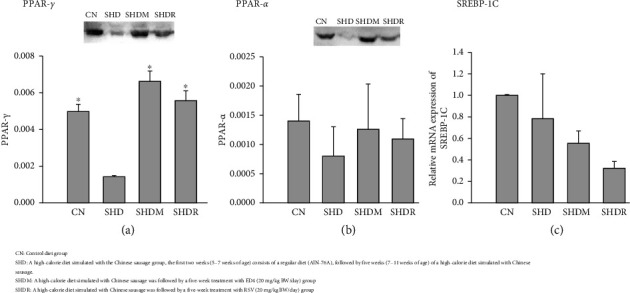
Effects on the lipid-regulated genes and protein expression. (a) PPAR-*γ*, (b) PPAR-α, and (c) SREBP-1C in the liver of Sprague–Dawley (SD) rats with obesity induced by a high-calorie diet simulated using Chinese sausage and then treated with resveratrol (RSV) and the purified monomer product 3-O-butanoylresveratrol (ED4).

This study showed that the SHD group exhibited the weakest levels and expression of PPAR-γ (Figure 4(a)) (*p* < 0.05). On the contrary, the SHDM and SHDR groups showed elevated and more abundant levels than the CN group following treatment. These phenomena imply that both ED4 and RSV can effectively regulate the level of PPAR-γ in the adipogenic pathway of rats and enhance adipose metabolism, among which ED4 was more effective. In addition, it was verified by the report of Shelton et al. [32] that mice fed a high-fat diet exhibited a pronounced decrease in PPAR-γ gene expression and protein abundance, which means depletion of PPAR-γ in the small intestinal epithelium, which was consistent with the findings reported in the present study. Another possible reason that may be attributable to the susceptibility of PPAR-γ to insulin is a principal and well-recognized regulator of adipogenesis, which functions in mitigating inflammation, glucose metabolism, fatty acid storage, lipolysis, and metabolism, modulating obesity, particularly by the sympathetic nervous system signaling [51, 52]. It has been reported that PPAR-γ serves as a pivotal regulator of the initial effects of high-fat dietary feeding on mucosal defenses in mice [52]. It has been reported that *Ligilactobacillus murinus* in *Lactobacillus* spp. regulates lipid secretion in intestinal epithelial cells by activating PPAR-γ in vivo and in vitro modes [32]. Concretely, butyrate metabolized by gut microbiota inhibits inducible nitric oxide synthase (iNOS) synthesis in the gut by modulating PPAR-γ signaling in colonic epithelial cells [53]. Therefore, the present study shows that these changes in hepatic lipid metabolism in SD rats by high-calorie diet model simulation using Chinese sausage are consistent with the previously reported severe decrease of PPAR-γ in intestinal epithelial cells caused by high-fat diet, subsequently leading to increased TG levels [32]. This also suggests that targeting PPAR-γ regulation could be a promising approach to modulating adipocyte development and lipid metabolism and alleviating metabolic dysfunction [32].

Regarding the PPAR-α levels, no significant differences were found in each group (Figure 4(b)), while the SHDM and SHDR groups were similar to the CN group regarding values. Remarkably, the SHDM group treated with ED4 achieved a favorable outcome. These phenomena also suggested that both ED4 and RSV could increase the expression of PPAR-α and its role in lipid metabolism to decrease the level of VLDL, preventing the excessive accumulation of lipids in blood vessels, thereby potentially mitigating the risk of cardiovascular disease. In addition, it has been reported that SCFA by the intestinal microbiota activates the AMPK pathway in lipid metabolism, while PPAR-α in the liver and brown adipose tissue initiates the expression of metabolic regulation, namely, adipogenesis has been shown to enhance the regulation of β-oxidation and metabolism of fatty acids [26, 36, 52, 54]. Interestingly, patients diagnosed with primary hypercholesterolemia, mixed dyslipidemia, and hypertriglyceridemia have been administered a synthetic fibrate PPAR-α agonist as part of their treatment [52]. Moreover, it has also been indicated that *A. muciniphila* plays a pivotal role in modulating the energy-appetite balance, contributes to the management of obesity, and mitigates inflammation through diverse mechanisms (enhancing the integrity of the intestinal epithelial barrier) [55], and hydrolyzes mucin, thereby creating a symbiotic environment for mutualistic gut microbiota [56], namely, promoting immune regulation and propionate production [57].

In the mRNA expression of SREBP-1C (Figure 4(c)), this study revealed that the CN group was the highest, while the SHD group was the second highest, but there was no statistical significance in all the groups compared to the SHD group. It is worth mentioning that the SHDM and SHDR groups contributed to a trend of decreased mRNA expression of SREBP-1C following five consecutive weeks of ED4 and RSV administration, which was the lowest in the SHDR group. These phenomena can be attributed to the fact that ED4 and RSV suppress the TG synthesis pathway, decreasing fat synthesis and effectively controlling BD gain. In addition, SREBP-1C has been reported to be a critical adipokine that regulates TG and fatty acid synthesis and accelerates fatty acid β-oxidation [26]. Duan et al. [26] also reported that high-fat diet-induced rats supplemented with fermented *Eucommia ulmoides* leaves extract for 8 weeks exhibited an up-regulation of PPAR-α expression that could initiate carnitine palmitoyltransferase 1A to promote fatty acid oxidation. In contrast, the mRNA level of the fatty gene SREBP-1c was markedly depressed, while these phenomena all agreed with the results of the present study. The same authors indicated that these benefits promoted bile acid synthesis and metabolism, decreasing TC and inhibiting lipid synthesis. Interestingly, bile acids have been shown to influence the structure and functionality of the gut microbial community [58, 59], while the composition of bile acids is subject to modifications through the bile salt hydrolases produced by the gut microbial community (over 2400 species) [59]. These pathways are also influenced by external factors (diet and antibiotics) [58]. However, more in-depth and extensive studies were required to confirm other detailed biosynthetic and metabolic pathways and mechanisms.

### 3.5. Effects on the Intestinal Flora

#### 3.5.1. Abundances and Diversities

Recently, high-throughput and NGS advancements have emphasized exploring the correlation between chronic diseases and gut microbiota dysbiosis [44]. However, it is imperative to acknowledge that the gut microbiota of individuals exhibits substantial diversity and can exhibit variations influenced by factors such as dietary patterns [50].

**Figure 5:**
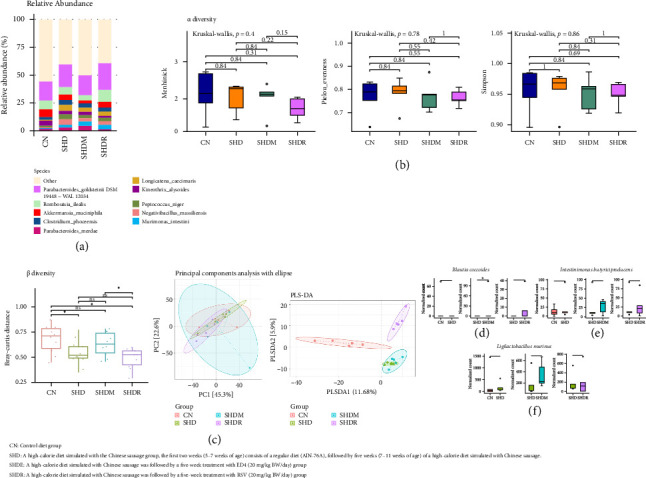
Effects on the intestinal flora of stool. (a) Relative abundance, (b) α diversity, (c) β diversity, (d) *Blautia coccoides*, (e) *Intestinimonas butyriciproducens*, and (f) *Ligilactobacillus murinus* in Sprague–Dawley (SD) rats with obesity induced by a high-calorie diet simulated using Chinese sausage and then treated with resveratrol (RSV) and the purified monomer product 3-O-butanoylresveratrol (ED4).

This study on the abundance of intestinal flora of obese SD rats showed that induction of SD rats using a high-calorie diet model simulation using Chinese sausage led to *Parabacteroides merdae*, *Murimonas intestini, Negativibacillus massiliensis*, *Peptococcus niger*, *Longicatena caecimuris*, *C. phoceensis*, and *P. goldsteinii* DSM 19448 (Figure 5(a)). However, SHDM and SHDR groups treated with ED4 and RSV for five consecutive weeks differentially promoted the proliferation of *P. goldsteinii* DSM 19448, *R. ilealis*, *A. muciniphila*, *M. intestini*, and others, and inhibited the proliferation of *C. phoceensis*, *L. caecimuris*, *P. niger*, *N*. *massiliensis*, and *P. merdae*. Concretely, ED4 and RSV differentially recovered the intestinal flora of SD rats and approached the CN group, induced substantial differences in species abundance, or promoted the proliferation of the specific strains mentioned above; that is, these phenomena were similar to the published findings [4, 27]. Moreover, this study showed a different abundance and distribution of intestinal flora compared to a study in which diets were introduced into a sliced cooked ham model in male Fischer 344 (F344/DuCrl) rats [60]. The same authors further indicated that the reduction in *Romboutsia* strains was correlated with a decline in *Peptostreptococcales*-*Tissierellales* abundance or intestinal injury in rats, which were associated with an increase in inflammatory cytokines and iNOS, formation of reactive oxygen species, etc., thereby increasing the risks for colon cancer and atrophy of skeletal muscle development [61]. Notably, the distribution and abundance of intestinal flora in the SHD group of this study differed from the results reported by Crowe et al. [21], while in the species, this study showed the opposite trend to the *Romboutsia* of the above research, and the *Akkermansia* exhibited a decreasing trend in both cases. Interestingly, the findings of increased abundance of *Clostridium* in the SHD group in the present study align with the abovementioned study, where diets containing either meat or sausage were linked to the proliferation of this species. Namely, nitrite has been reported to prevent the growth of *Clostridium* spp. [21], the three groups of rats consuming sausage-containing ingredients in this study showed increased *Clostridium* spp. compared to the CN group. However, the absence of nitrite does not constitute a factor interfering with the disparities in gut microbiota in this study. Although the same ratios of modifying diet recipes were used (described in Section 3.1), other factors that might have contributed to the difference were the varying experimental animal strains and durations of trials. It has been reported that mice induced by a Western diet (Open Source Diet Formulation D12451) and then converted to a low-fat diet (Open Source Diet Formulation D12450B) presented a remarkably higher relative abundance of *Akkermansia* with or without saline supplementation [24]. Moreover, it has been reported that treating high-fat-diet–induced obesity in mice with polyisoprenylated benzophenone derivative isolated from *Garcinia indica* mitigates inflammation by increasing the intestinal symbiotic bacterium *Akkermansia* [62]. Therefore, the clinical study also reported that intestinal *Akkermansia* was associated with the clinical status of host health, implying a decrease in intestinal *Akkermansia* abundance in obese patients [55], a tendency consistent with the present findings. Multiple research studies have also indicated that dietary polyphenols or flavonoids can contribute to the increased abundance of *Akkermansia* spp. in the rodent model intestinal tract [62–64]. Interestingly, it has been reported that alterations or dysbiosis of the gut microbiota caused by antibiotic exposure and high-fat diets possibly disrupt the activity of intestinal epithelial cells, thereby contributing to obesity, particularly in the pediatric population [32].

Moreover, oral butyrate supplementation has enhanced energy expenditure, ameliorated insulin sensitivity, and reduced BW in the high-fat rodent model [65]. In subjects with metabolic syndrome compared to lean individuals, butyrate supplementation has been observed to influence serum lipid composition and gut microbiota, thereby regulating BW and lipid metabolism [66]. Notably, it has been reported that *Clostridium butyricum* adversely affects high-fat-diet-induced intestinal inflammation, which was more pronounced in male Fischer-344/NSIc rats than in females, whereas *C. butyricum* also increased stool butyric acid concentrations [67]. However, it differed from the present study in that the SHD group in this study exhibited higher *C. phoceensis* abundance. In contrast, the SHDM and SHDR groups followed (Figure 5(a)), except that the butyric acid content in blood was higher than in stools. In particular, SHDM contained the highest amount of butyric acid in the blood, while SHDR contained the lowest amount in stools. The present study showed that stools' acetic and butyric acid contents were negatively correlated with the abundance of *A. muciniphila*, consistent with the phenomenon reported by Choi et al. [67] and Li et al. [47].

Regarding α diversity, this study showed that all groups were not significantly worse in Menhinick, Pielou's evenness, and Simpson's analysis (Figure 5(b)). Consequently, we hypothesized that supplementation with either ED4 or RSV does not adversely affect intestinal flora diversity compared to antibiotics or pharmaceuticals, consistent with numerous published reports [27, 28, 32]. It has also been reported that diets containing Frankfurter sausages decreased the microbial diversity of the mouse intestinal tract, leading to intestinal dysbiosis, in which the population of *Dubiosiella Newyorkenesis* is dominant [21], but the same authors have confirmed that this strain is not involved in the production of TMAO. The distribution patterns of gut microbiota may provide valuable insights into the etiology, progression, and potential reversal of obesity, although dietary modifications, particularly those related to fat content, appear to influence the composition of the gut microbiota [24]. Therefore, this study showed that the SHDM group administered with ED4 exhibited a more satisfactory performance than the SHDR group (administered RSV) in terms of abundance or homogeneity of the flora. Consequently, ED4 supplementation may positively affect the diversity of the gut microbiota in high-fat diets. The results of this study revealed β diversity (Figure 5(c)) among the groups in the Bray-Curtis matrix showed a significant difference (*p* < 0.05) for both SHD and SHDR groups compared to the CN group. It is worth mentioning that there was no difference between the SHDM and CN groups. Despite no significant differences between the SHDM and SHDR groups compared to the SHD group, it was found that the species diversity was higher in the SHDM and SHDR groups compared to the SHD group. Moreover, there was a significant difference between the SHDM and SHDR groups (*p* < 0.05), which implies that the ED4 treatment administered by the SHDM group provided the best recovery of flora nearest to the CN group for species diversity. Next, the results of the Principal Coordinate Analysis (PCoA) using Ellipse demonstrated low dispersion and considerable overlap in the datasets. This indicated a high degree of similarity in the composition of the microbial communities across all groups, with the ED4 treatment group showing the broadest distribution of bacterial flora. These findings regarding the regional clustering of gut microbiota across groups are consistent with the results reported by Wen et al. [68]. However, compared to those mentioned in previously published reports, α- and β-diversity did not differ significantly but produced significant differences in intestinal microbiota abundance, and specifically, the abundance of *Akkermansia* also showed an opposite trend. However, these variations and disparities can be ascribed to differences in the models used for diet offerings. Specifically, a previous study employed a high-fat diet consisting of 3.5% cholesterol, 0.5% sodium cholate, 0.2% propylthiouracil, 5% sugar, 10% lard, and 80.8% basal diet [68]. Remarkably, this phenomenon in this study follows the results of a previously published report: RBE treatment significantly altered the microbial community [16], and ED4 was a single substance obtained by isolation and purification from RBE. While the diversities were not significantly different, the three strains containing *Parabacteroides* spp., *Intestinimonas*, and *Akkermansia* were all detected. The other differences were still variations in the administration conditions and dietary composition. However, in the PLS-DA analysis, the SHD group exhibited a reduced distribution of colonies and demonstrated significant overlap with the SHDM group. In addition, there was an increased distribution of populations in the SHDM and SHDR groups administered ED4 and RSV treatments compared to the SHD group. All results, such as the distribution of bacterial abundances and diversities in the intestinal microflora of stool, were compared with the published results of our team [4], and all showed similar trends and therapeutic efficacy despite using different obesity-inducing models. In particular, a total of eight strains were detected in the TOP10 bacterial abundance, including *P. merdae*, *M. intestini, P. niger*, *Kineothrix alysoides*, *L. caecimuris*, *A. muciniphila*, *R. ilealis*, and *P. goldsteinii* DSM 19448. Therefore, it also implied that this study's high-calorie diet model simulation using Chinese sausage to induce obesity in SD rats confirmed that results in line with the traditionally used high-fat model could be obtained. However, the results and limitations of this study may be attributed to differences in exercise capacity between rats within the same group or nearly uniform interactions between their gut floras. To mitigate this, future studies could house one rat per cage, ensuring sufficient space for exercise and eliminating mutual influence on each other's microbiota.

#### 3.5.2. Individual Strain

Regarding individual strains, this study showed a significant difference (*p* < 0.05) in the SHDM group compared to *Blautia coccoides* in the SHD group (Figure 5(d)). Yet, in *L. murinus* and *Intestinimonas butyriciproducens* compared to the SHD group, all groups were without significant differences (Figure 5(e) and 5(f)). Interestingly, Shelton et al. [32] reported that decreasing *L. murinus* in the small intestinal microbiota of mice can increase fat accumulation with short-term microbiota alterations leading to long-lasting metabolic changes, in particular in early life mice reared on a high-fat diet and exposed to low-dose penicillin (6.67 mg/L). That is, *L. murinus* confers protection against obesity in juvenile mice subjected to a high-fat diet [32]. The same authors also reported that *Lactobacillus* spp. (in vitro) showed hypersensitivity to combinations of penicillin and lipids or penicillin and bile salts, which were elevated in the intestinal tract when reared on a high-fat diet (in vivo), and this finding agreed with the results of the present study. Specifically, the SHD group exhibited the lowest levels of *L. murinus* in this study, although no statistically significant difference was observed compared to all other groups. In addition, it has been reported that *Intestinimonas* can produce SCFA, such as butyrate, within the intestinal tract [43]. The positive effects of ED4 and RSV supplementation, which help restore the intestinal flora in obese rats disrupted by high-calorie diets and physiological imbalances (as described in Sections 3.1–4), contribute to promoting overall wellness. The limitations of the present study are the existing limitations in sampling gut microbiota. In future research, it is vital to consider the practicality and feasibility of safely and noninvasively collecting, characterizing, and quantifying gut microbiota, metabolome, and bile acids throughout the intestinal tract of SD rats during normal digestion. This aspect was also highlighted in similar reports by Li et al. [69] and Shalon et al. [70].

## 4. Conclusions

This study revealed that five consecutive weeks of supplementation with ED4 and RSV effectively managed BW and recovered the intestinal flora to a certain extent akin to normal levels in high-calorie diet model simulation using Chinese sausage-induced obese SD rats, particularly ED4, which performed satisfactorily. Previous findings by our team have indicated that the ED4 (monoester) form of RBE is more effective in preventing obesity, and this was again confirmed in this study with Chinese sausage as the food base in the high-calorie model. However, potential system alterations could arise because of mere dietary inclinations. Specifically, the anti-obesity effect of ED4 was observed through its influence on gut microbiota composition concerning alterations in SCFA levels. Conversely, further investigation is required to comprehend and fully clarify the intricate mechanisms underlying these interactions. Despite the promising results from the present study conducted on rodent models, subsequent research should prioritize translating these findings into clinical studies and concurrently assessing the potential efficacy and food safety of incorporating ED4 as a food additive. Moreover, this study presents a potentially more consumer-friendly alternative for body weight and health management in addition to the existing options of dietary supplementation such as herbal extracts, probiotics, and prebiotics.

## Figures and Tables

**Table 1:** Effects on the short-chain fatty acid (SCFC) contents of serum and stool in Sprague–Dawley (SD) rats with obesity induced by a high-calorie diet simulated using Chinese sausage then treated with resveratrol (RSV) and the purified monomer product 3-O-butanoylresveratrol (ED4).

Group	Serum				Stool			
	Acetic acid	Propionic acid	Isobutyric acid	Butyric acid	Acetic acid	Propionic acid	Isobutyric acid	Butyric acid
	(μM)				(μM)			
CN	1528.48 ± 217.38	47.88 ± 7.66	18.08 ± 0.71	41.07 ± 9.87	1535.03 ± 671.59	68.85 ± 2.27	46.91 ± 4.57	50.91 ± 4.27
SHD	802.14 ± 96.53^∗^	47.15 ± 4.90	13.58 ± 1.56^∗^	46.19 ± 14.27	6897.30 ± 831.45^∗^	53.19 ± 1.80^∗^	31.85 ± 1.29^∗^	41.89 ± 1.54
SHDM	1121.31 ± 134.36^∗^	57.73 ± 6.34^∗^	16.40 ± 0.60	56.66 ± 5.89^∗^	6993.35 ± 862.74^∗^	61.69 ± 2.28	31.58 ± 0.59^∗^	41.46 ± 1.26^∗^
SHDR	1405.19 ± 280.11	55.62 ± 5.22^∗^	16.33 ± 0.37	49.12 ± 4.23^∗^	8373.23 ± 737.26^∗^	68.17 ± 3.37	30.49 ± 1.31^∗^	35.93 ± 1.79^∗^

*Note:* The groups are abbreviated as follows: CN (control group): control diet group; SHD: a high-calorie diet simulated with the Chinese sausage group, the first 2 weeks (5–7 weeks of age) consists of a regular diet (AIN-76A), followed by 5 weeks (7–11 weeks of age) of a high-calorie diet simulated with Chinese sausage; SHDM: a high-calorie diet simulated with Chinese sausage was followed by a 5-week treatment with ED4 (20 mg/kg BW/day) group; SHDR: a high-calorie diet simulated with Chinese sausage was followed by a 5-week treatment with RSV (20 mg/kg BW/day) group.

^∗^ *p* < 0.05 compared to CN.

## Data Availability

The data that support the findings of this study are available from the corresponding author upon reasonable request.

## Supporting Information

The supporting informations associated with this study provide detailed background information supporting the methodology and results.

Table A1 presents the formulation of the Chinese sausage used to simulate a high-calorie diet, including its composition and ingredients.

Table A2 summarizes the experimental chow diets' nutritional composition and caloric content, contrasting the control (AIN-76A) and the modified sausage-based diets.

Table A3 lists the specific primer sequences used in the real-time PCR analysis for gene expression studies.

Table A4 describes the thermal cycling conditions employed in the real-time PCR assay.

Table A5 reports the physiological parameters observed in the animal model, including feed conversion rate (FCR), specific growth rate (SGR), mortality, and relative organ weights in response to the different treatments.

All supporting tables are referenced in the manuscript and support the conclusions drawn in the physiological and molecular analysis.
