# Supplementary material for: Chinese Sausage Simulates High Calorie–Induced Obesity In Vivo, Identifying the Potential Benefits of Weight Loss and Metabolic Syndrome of Resveratrol Butyrate Monomer Derivatives
Source: J Nutr Metab. 2025 May 16;2025:8414627. doi: 10.1155/jnme/8414627 (PMC12101907; doi:10.1155/jnme/8414627)
Supplement: Supporting Information — Additional supporting information can be found online in the Supporting Information section. [file 8414627.f1.docx]

**Supplementary Materials**

Table A1 presents the formulation of the Chinese sausage used to simulate a high-calorie diet, including its composition and ingredients.

Table A2 summarizes the experimental chow diets' nutritional composition and caloric content, contrasting the control (AIN-76A) and the modified sausage-based diets.

Table A3 lists the specific primer sequences used in the Real-time PCR analysis for gene expression studies.

Table A4 describes the thermal cycling conditions employed in the Real-time PCR assay.

Table A5 reports the physiological parameters observed in the animal model, including feed conversion rate (FCR), specific growth rate (SGR), mortality, and relative organ weights in response to the different treatments.

| Table A1. Composition of Chinese sausage recipe | | |
| --- | --- | --- |
| Content | | Quantity (g) |
| Pork | Lean meat | 764 |
|  | Back fat | 191 |
| Basic Seasonings | Salt | 25 |
|  | Sugar | 20 |
| Food Grade Additives | Nitrite | 0.025 |
|  | | |

| Table A2. The composition, primary nutrient content, and calorie contents of the modified chow diets for experimental animals | | |
| --- | --- | --- |
| Nutritional Composition | Control diet  (CN) | Chinese Sausage simulates a high-calorie diet  (SHD) |
|  | Purified rodent diet American Institute of Nutrition (AIN)-76A 100% | AIN-76A/Chinese sausages powder 85/15% (*w/w*) |
| Carbohydrate (%) | 65.20 | 65.70 |
| Fat (%) | 5.10 | 6.50 |
| Protein (%) | 18.10 | 19.20 |
| Calories (kcal/ g) | 3.79 | 3.98 |

| Table A3. Specific primers used in Real-time PCR in this study | |
| --- | --- |
| Gene (rat) | Primer sequence (5ʹ–3ʹ) |
| SREBP-1C | F-ACCGTTCCTCTATCAATGACAAGAT  R- TGTGCTGTAAGAAGCGGATGTAGT |
| R18S | F- GCCGCGGTAATTCCAGCTCCA  R- CCCGCCCGCTCCCAAGATC |
| F: Forward primer. R: Reverse primer. | |

| Table A4. Real-time PCR conditions used in this study | | | |
| --- | --- | --- | --- |
| Program | Temperature (°C) | Time | Number of cycle |
| 1 | 95 | 3 min | × 1 |
| 2 | 95 | 10 sec | × 45 |
|  | 60 | 50 sec |  |
|  | 72 | 5 sec |  |
| 3 | 95 | 5 sec | × 1 |
|  | 65 | 1 min |  |
|  | 97 |  |  |

| Table A5. Effects on various physiological parameters, including mortality, feed conversion rate (FCR), specific growth rate (SGR), and relative organ weight in Sprague-Dawley (SD) rats with obesity induced by a high-calorie diet simulated using Chinese sausage then treated with resveratrol (RSV) and the purified monomer product 3-O-butanoylresveratrol (ED4) | | | | | |
| --- | --- | --- | --- | --- | --- |
| Item | | CN | SHD | SHDM | SHDR |
| Mortality (%) | | 0 | | | |
| FCR (%) | | 0.16 ± 0.01 | 0.12 ± 0.02 | 0.09 ± 0.01* | 0.13 ± 0.01 |
| SGR (%) | | 0.36 ± 0.01 | 0.39 ± 0.02 | 0.33 ± 0.02 | 0.36 ± 0.01 |
| Organ relative weight  (%) | Liver | 3.58 ± 0.07 | 3.44 ± 0.15 | 3.72 ± 0.17 | 3.70 ± 0.17 |
|  | Kidney | 0.45 ± 0.01 | 0.39 ± 0.01 | 0.42 ± 0.01 | 0.42 ± 0.01 |
|  | Epididymis | 3.20 ± 0.55 | 6.65 ± 0.72* | 3.14 ± 0.40 | 3.81 ± 0.43 |
|  | Retroperitoneal | 4.35 ± 0.38 | 8.75 ± 1.23* | 4.81 ± 0.28 | 4.26 ± 0.53 |
| The groups are abbreviated as follows: CN (Control group): Control diet group, SHD: A high-calorie diet simulated with the Chinese sausage group, the first two weeks (5–7 weeks of age) consists of a regular diet (AIN-76A), followed by five weeks (7–11 weeks of age) of a high-calorie diet simulated with Chinese sausage, SHDM: A high-calorie diet simulated with Chinese sausage was followed by a five-week treatment with ED4 (20 mg/kg BW/day) group, SHDR: A high-calorie diet simulated with Chinese sausage was followed by a five-week treatment with RSV (20 mg/kg BW/day) group. FCR (%): (6–10 weeks food intake/10 week BW) ×100. SGR (%): (BW of 10 week－6 week/10 week BW) × 100. Relative kidney weight (%): calculated as the abdominal right kidney weight divided by BW multiplied by 100. Relative liver weight (%): calculated as the abdominal liver weight divided by BW, multiplied by 100.  * *p*< 0.05 compared to CN. | | | | | |
